# Perceived feasibility and acceptability of an innovative emotion regulation programme with physical activity elements for older South African adolescents from low-income settings: a qualitative study

**DOI:** 10.1186/s12887-025-06280-6

**Published:** 2025-11-11

**Authors:** C. Ward-Smith, K. Sorsdahl, M. Berking, C. van der Westhuizen

**Affiliations:** 1 Department of Psychiatry & Mental Health, Alan J. Flisher Centre for Public Mental Health (CPMH), University of Cape Town, Cape Town, ZA South Africa; 2 University of Erlangen-Nuremberg, Erlangen, Germany

**Keywords:** Adolescent mental health, Emotion regulation, Mental health interventions, Depression, Anxiety

## Abstract

**Background:**

Adaptive emotion regulation and physical activity may protect against mental health conditions such as depression and anxiety in adolescents. However, contextually relevant psychosocial interventions remain scarce in South Africa, and limited research explores adolescents’ lived experiences and perceived impacts of these interventions. This qualitative study examined the feasibility, acceptability, and perceived impact of a co-adapted, task-shared emotion regulation intervention incorporating physical activity.

**Methods:**

The group-based programme was piloted in a single-arm feasibility study with 85 adolescents (aged 15–18) across four low-income schools in the Western Cape. Semi-structured interviews (40–60 min) were conducted with adolescents (*n* = 20), community stakeholders (*n* = 7), and facilitators (*n* = 4) to explore their perceptions of the intervention.

**Results:**

Five key themes emerged: (1) Perceived programme acceptability, (2) Experiences with training and supervision, (3) Barriers and facilitators to delivery and retention, (4) Perceived impact on mental health and (5) Facilitators of learning. Participants found the intervention engaging and relevant, highlighting its role in strengthening emotion regulation skills. Adolescents reported improved mood and behaviour, with reductions in stress, anger, anxiety, and depressive symptoms, alongside enhanced academic engagement and social connectedness. Facilitators viewed the task-sharing model, supported by competency-based training and supervision, as comprehensive and valuable for successful implementation. Experiential learning, enjoyment, and the use of digital and hardcopy materials were found to be key facilitators of learning in the programme.

**Conclusions:**

The findings highlight the feasibility and acceptability of integrating emotion regulation skills with creative, movement-based strategies in adolescent mental health interventions. By combining emotion regulation training with physical activity in an engaging group setting, the programme promoted adaptive emotional coping strategies. This study emphasizes the potential of creative, experiential, evidence-based approaches to address adolescent mental health needs in low-resource settings. This feasibility study was registered retrospectively with the Pan African Clinical Trial Registry (PACTR202412659160564, registered 17/12/2024). This study can be accessed at: https://pactr.samrc.ac.za/Search.aspx.

**Supplementary Information:**

The online version contains supplementary material available at 10.1186/s12887-025-06280-6.

## Introduction

Adolescence is a critical period of vulnerability to mental health conditions (MHCs), which, if untreated, can profoundly impact health and life trajectories [1, 2]. Globally, approximately one in seven 10–19-year-old adolescents experience a MHC, accounting for about 13% of the global burden of disease in this population [3, 4]. Depression, anxiety and substance use conditions are the leading cause of disability in adolescents, accounting for approximately 24.9% of all years lived with disability (YLDs) [3, 5–8]. In sub-Saharan Africa, approximately 27% of adolescents report depression symptoms, 30% anxiety, and 41% emotional or behavioural problems^1^ [9]. Though limited, South African studies report concerning levels of MHCs among adolescents [10–18]. Cross-sectional data indicates that 56.9% of older adolescents are at risk for anxiety, 57.7% for depression, and 31.4% for PTSD symptoms [19]. Despite these statistics, there are significant treatment gaps for local adolescents. Adolescents aged 10–19 constitute approximately 23% of the population, underscoring the need for targeted and creative mental health (MH) interventions to address their unique challenges and mitigate the long-term impacts of untreated MHCs [20].

School-based prevention interventions show promise in addressing mental health (MH) gaps by engaging adolescents in accessible educational settings [21, 22]. These interventions equip adolescents with tools to manage emotional distress and prevent common MHCs. Systematic reviews and guidelines advocate for such interventions, emphasizing evidence-based components like emotion regulation (ER) skills, physical activity, cognitive reframing, problem-solving, and self-compassion—each shown to improve MH outcomes for adolescents [23–27]. Integrating physical activity into ER interventions is particularly promising due to its creative, experiential appeal, its ability to reduce anxiety and depression symptoms, and its enhancement of emotional flexibility, thus complementing ER strategies [28–30]. Emotional dysregulation, a key factor associated with depression and anxiety [31–38], is particularly prevalent among 12–15-year-olds, who are vulnerable to maladaptive ER strategies [39, 40]. ER-focused interventions, especially those delivered by non-specialists, have been linked to improved emotional management and long-term MH outcomes for adolescents [27, 41–43]. Qualitative findings support these outcomes, with adolescents highlighting improved stress and ER, a sense of belonging fostered through group activities, and the importance of contextually relevant materials and content [43–45]. Physical activity interventions for adolescents have been shown to enhance self-efficacy, foster social cohesion, and provide an outlet for emotional expression [46–48], while also offering neurobiological benefits, such as reduced cortisol levels and improved executive functioning, thereby complementing ER in school-based mental health prevention [46, 47].

In response to the need for accessible, creative and contextually appropriate MH interventions, this study co-adapted an evidence-based ER intervention called *Affect Regulation Training* (ART) [42, 48]. *ART* has shown promise as a stand-alone ER programme for adults and adolescents, in community and clinical populations, across a broad range of MHCs [34, 49–52]. *ART* has been adapted for use among young Belgian adolescents with clinical obesity [53] and young Belgian adolescents in school-settings [54–56]. The locally co-adapted version of this programme, known as the #FeelThinkMove Programme (#FTM), integrates innovative physical activity elements and aims to equip South African adolescents aged 15 to 18 with mental, emotional, and physical skills to manage emotional distress.

The #FTM Programme integrates evidence-based ER skills—such as Relaxation, Awareness, Acceptance, Self-Support, Investigation (exploring how situations, feelings, thoughts, and behaviours are linked), Distraction, Problem-Solving, and Thinking-Differently—with flexible physical activity elements, including cardio and strength exercises, ball games, and dance activities. Previous quantitative research demonstrated the #FTM Programme's feasibility, acceptability, and therapeutic promise for older adolescents in low-income settings experiencing symptoms of depression and anxiety. Qualitative data can offer deeper insights into how such interventions are experienced by adolescents. For instance, adolescents from low-and middle-income US settings who participated in a school-based mindfulness and ER programme reported improved stress management, ER, and a sense of community through group activities [44]. Further, South African adolescents from low-income settings who took part in a sports-based wellbeing programme reported experiencing an increased sense of belonging, protection from negative influences, physical and emotional benefits, access to positive role models, and gaining valuable life skills, which were perceived as fostering overall well-being and resilience [57]. Yet, few studies explore how adolescents in low-resource settings experience ER and physical activity interventions, and how these interventions can be optimized for real-world implementation [45, 58]. Furthermore, few studies examine such interventions from the perspectives of multiple stakeholders, including adolescents, intervention facilitators, and community members, particularly for task-shared approaches in low-income settings. This study aimed to explore the perceptions of adolescents, community stakeholders and facilitators regarding the feasibility, acceptability, and perceived impact of the #FTM Programme—a school-based, task-shared, ER and physical activity intervention for 15- to 18-year-old South African adolescents from low-income settings experiencing symptoms of depression and/or anxiety.

## Methods

This descriptive, qualitative study reported according to the Consolidated Criteria for Reporting Qualitative Research (COREQ) (Appendix 1) [59]. Ethical permission was granted by the Human Research Ethics Committee at the University of Cape Town (#558/2021). All participants provided written informed consent to join the study. Adolescents under 18 years completed written informed assent, along with informed consent from their caregivers.

### Setting and study sites

This study was conducted in 4 high schools in the greater Cape Town, Winelands, and Southern Peninsula areas of the Western Cape, South Africa. These schools provide education for learners from low-income backgrounds and fall within the Western Cape Education Department’s 1–3 quintiles schools, which means that they are ‘no-fee’ or ‘low fee’ paying schools. Schools were identified through engagement with an non-governmental organisation, Community Keepers (CK)^2^, which provides psychosocial support to learners at approximately 80 designated schools throughout South Africa. Ethical permission was granted by the Western Cape Education Department to collaborate with these schools.

### Participants

The study included 31 participants: purposively selected adolescents who completed > 50% of the #FTM intervention (*n* = 20), intervention facilitators (*n* = 4), and community stakeholders (*n* = 7). The adolescents were originally recruited into the main feasibility study if they had scores ≥ 10 on the Patient Health Questionnaire for Adolescents (PHQ-A) and/or Generalized Anxiety Disorder 7-item (GAD-7)^3^ [60, 61]. Note that both the PHQ-A and GAD-7 are in the public domain and may be freely used for non-commercial clinical or research purposes without permission [62, 63]. Adult participants included facilitators (all female, 3 Registered Counsellors, 1 Social Worker) and stakeholders (1 female teacher, 2 male principals, and 4 CK staff members—1 female Social Worker, 3 female Registered Counsellors). All participants in this study voluntarily opted to participate, and none declined. The sample size aligns with thematic saturation recommendations [64–66].

### Procedure

At the three-month follow-up for the main feasibility study, 40–60-min semi-structured, face-to-face interviews were conducted with adolescents, facilitators, and community stakeholders to gather feedback for programme improvement. Adolescents were interviewed privately at their schools by female #FTM facilitators, all of whom were Registered Counsellors (RCs) or Social Workers (SWs) and unaffiliated with the adolescents’ programme delivery. The facilitators introduced themselves as #FTM researchers, explaining that their role was to gather feedback for programme refinement. Facilitators were interviewed at the research team’s offices by an independent researcher, while community stakeholders were interviewed privately at their schools by #FTM facilitators. Although community stakeholders were familiar with the facilitators, they had not worked directly with them. To ensure transparency, all interviewers introduced themselves, clarified their roles, and reiterated that the interviews aimed to gather feedback on the intervention programme and training. Facilitators and the lead researcher had no prior relationship with the adolescent participants, and the independent researcher was unknown to the facilitators. All interviews were guided by tailored interview guides (Appendix 2) and focused on programme improvement feedback. Interviews were audio-recorded with permission, and participants were reimbursed for transport (if needed), provided with snacks and juice, and given R150 grocery vouchers as a token of appreciation.

### Intervention

See Table 1 for the specific components of the #FTM intervention package, including facilitator characteristics, intervention structure, session content, physical activity elements, homework tasks, materials, training and supervision format and supervisor characteristics.

**Table 1:** Components of the #FTM intervention package

1. Facilitator characteristics	• Main facilitator: RC (> 2years experience) • Co-facilitator: SW or similar qualification (> 1 year experience + exposure to adolescent MH)
2. Structure of intervention package	• 7 sessions delivered weekly, after school, over 7 weeks; session length = 2 h each
3. Session structure and surveys:	
1. Baseline survey	• Screening and baseline MH survey conducted
2. Session 1	• Module 1: orientation; goal-setting; introduction to breathing and muscle *Relaxation* skills; introduction to *Awareness*; Gym Club
3. Session 2	• Module 2: recap; introduction to skills of emotional *Acceptance* and the function of emotions; Gym Club
4. Session 3	• Module 3: recap; introduction to 3 × *Self-support* skills: self-esteem, self-care, and positive self-talk; Gym Club
5. Session 4	• Module 4: recap, introduction to 5 × *Investigation* components: situations, feelings, thoughts, behaviours and consequences; Gym Club
6. Session 5	• Module 5.1: recap, introduction to *'Regulation'* and 2 × ER strategies: *Distraction* and *Problem-Solving;* Gym Club
7. Session 6	• Module 5.2: recap, introduction to ER strategy #3: *Thinking Differently;* Gym Club
8. Session 7	• Module 6: recap whole programme; content integration and consolidation; build an 'emotional toolkit.'; Gym Club
9. 8-week follow up survey	• 8-week follow-up MH survey conducted
10. 3-month follow up survey 4. Physical activity elements	• 3-month follow-up MH survey, programme evaluation survey and qualitative interviews with sub-sample of participants conducted • Weekly practice of Gym Club (mix of cardio and strength-based sequences of exercises) and/or physical activity games (dancing and ball skills)
5. Homework tasks	• Listening to weekly audio recording and complete a physical activity exercise (Gym Club or any other physical activity)
6. Digital and hardcopy materials	• Audio recordings, activity books, star breathing handout, magnetized puzzles as weekly nudges, psychoeducation videos
7. Programme training format	• 40 h formal training (5 days). Mixture of didactive teaching and experiential group activities (role plays)
8. Programme supervision format	• Additional 24 h of supervision over the course of the programme: 2 h of weekly, face-to-face supervision, debriefing and extra training as necessary. Facilitation competency was assessed during training role plays as well as after programme sessions using an adapted competency tool [67]. A programme-specific checklist (for each session) was used to assess content fidelity
9. Supervisor characteristics	• Clinical Psychologist registered with the HPCSA (conduct guided by professional standards and ethics), with > 5 years clinical experience; trained in CBT, ACT and IPT; specialized experience in working with adolescents

### Facilitator training, supervision, and competency monitoring

A task-sharing model adapted from prior South African research was used, integrating comprehensive training with ongoing and ad hoc supervision [68, 69]. Facilitators received 40 h of facilitator training on depression, anxiety, counseling skills, and the manualized intervention. Facilitator core competencies were assessed using the adapted Therapeutic Attributes and Competencies for Teen groups in South Africa (TACT-Group-SA tool^4^), rated by facilitators, the lead researcher, and an independent rater. TACT-Groups-SA facilitation was assessed across three domains: (1) group facilitation skills, (2) facilitator attributes, and (3) communication skills. Evaluations focused on facilitators’ ability to manage group dynamics, participation, and pacing, their demonstration of confidence, warmth, and teamwork, and their use of verbal and non-verbal communication to create a supportive and engaging environment. Quantitative results on facilitator competency are reported elsewhere; qualitative findings on training and supervision are provided here.

### Data analysis

Interview recordings were transcribed verbatim and anonymized by omitting names of participants and places. Data analysis was conducted using NVivo 14 and Braun and Clarke's six-phase Thematic Analysis [58, 70] a) familiarizing with data through active reading, b) generating initial codes, c) collating codes into themes, d) reviewing and refining themes, e) defining and naming themes, and f) producing the report.

The lead researcher (CWS) used a combined deductive and inductive approach to derive themes, developing an initial coding framework informed by research objectives while allowing unexpected themes to emerge from the data for a nuanced analysis [71]. This framework was refined collaboratively with the research team (KS and CvW). Each team member independently reviewed and coded a sub-sample of transcripts (*n* = 4) using the framework. Intercoder reliability was strong (Cohen’s Kappa, κ = 0.84).

## Results

Table 2 presents an overview of adolescent participants’ gender, age, baseline depression and anxiety symptom scores [60, 72], ER scores [73] as well level of engagement in the programme.

**Table 2:** Adolescents’ sociodemographic information, level of programme engagement and MH characteristics (*n* = 20)

Variable	n (%)	Females	Males
	**-**	*n* = 14	*n* = 6
**Age m(sd)**	16.30 (1.13)	16.21 (1.25)	16.50 (0.84)
15 years	7 (35.00)	6 (42.90)	1 (16.70)
16 years	3 (15.00)	2 (14.30)	1 (16.70)
17 years	7 (35.00)	3 (21.40)	4 (66.70)
18 years	3 (15.00)	3 (21.40)	0 (0.00)
**Average number of sessions completed m(sd)**	6 (1.46)	6 (1.66)	6.5 (0.84)
Engaged (completed > 75% of sessions)	15 (75.00)	10 (71.40)	5 (83.30)
Disengaged (completed < 75% of sessions)	5 (25.00)	4 (28.60)	1 (16.70)
**Depression symptoms (PHQ-A) m(sd)**	16.20 (4.95)	17.36 (4.89)	13.50 (4.28)
**Anxiety symptoms (GAD-7) m(sd)**	13.30 (4.08)	14.21 (4.39)	11.17 (2.32)
**Emotion regulation (DERS-16) m(sd)**	37.35 (12.24)	39.57 (12.69)	32.17 (10.23)

### Overview of themes

Four main themes were identified: 1) Perceived programme acceptability, 2) Perceptions of training and supervision 3) Perceived barriers and facilitators to programme delivery and retention 4) Perceived impact of the programme and 5) Facilitators of learning. See Table 3 for a summary of the main themes and subthemes (coding tree). Please note the following key for the in-text participant references. CS = community stakeholder, RC = registered counsellor, SW = social worker, P denotes an adolescent participant, F = female, M = male.

**Table 3:** Summary of main themes and subthemes

Main themes	Subthemes
1. Perceived programme acceptability	1. Perceived need for ER skills 2. Perceived appropriateness of programme for school settings 3. Acceptability of #FTM facilitators and facilitator characteristics
2. Perceptions of training and supervision	
3. Perceived barriers and facilitators to programme delivery and retention	1. School engagement 2. Motivation for programme attendance and MH stigma 3. Logistical factors for programme delivery and retention
4. Perceived impact of the programme	1. Positive shifts in mood and behaviour 2. Salient ER skills and perceived benefits 3. Additional benefits
5. Perceived facilitators of learning	1. Experiential learning and the importance of having fun 2. #FTM digital and hardcopy materials

### Perceived programme acceptability

This theme describes participants’ perceptions of programme acceptability overall. Sub-themes here include the perceived need for ER skills; appropriateness of the programme for school settings and acceptability of facilitators and facilitator characteristics.

#### Perceived need for ER skills

Stakeholders and many adolescents emphasized the need for emotional education in schools, and the importance of teaching ER skills in these settings. A school principal highlighted students' lack of emotional awareness: *“[…] some learners are unaware of the underlying challenges they are facing… suppressing their feelings …"* (CL5, M). Similarly, P3 (15, F) noted that some teenagers only recognize their need for support when they reach a crisis point: *“…[some] can’t accept that they need help…they only notice…at the latest stage…”* Many community stakeholders also observed signs of emotional dysregulation, with reports of adolescents experiencing anxiety and panic attacks but lacking coping skills. Further, facilitators and most adolescents noted that teenagers may resort to maladaptive coping strategies, like using alcohol or engaging in self-harm, when facing emotional struggles.

Many participants also noted perceived gaps in taught ER skills in school settings. Here, CS5 (SW, F) noted, *"They'll teach you about everything else, but except how to regulate your emotions […] nobody teaches you those things, so it would be great if more schools could have it.”* Adolescents echoed this sentiment and stressed the need for including ER skills in school curriculum to provide teenagers with stress management skills. P12 (aged 15, M) noted, *"If a school had to incorporate these lessons, most students would actually have a coping mechanism… in case they're in serious stress… I think these skills are very helpful."* Others noted how the programme had addressed this gap, with CS6 (SW, F), sharing, *"You gave them the tools to manage emotional distress… It's amazing to hear the benefits."* Many adolescents also appreciated the learned skills, expressing the desire for wider access for others. P15 (15, F) expressed, "*If I didn't come, I wouldn't have known… I wish everyone could know the skills we learned…because if you check how many kids that don't know, there's a lot of them."*

#### Perceived appropriateness of programme for school settings

Overall, all participants expressed a strong sense of the programme's acceptability. Facilitators highlighted personal benefits, its appeal to adolescents, and the well-structured content. One facilitator shared, *“… It’s a great programme. I've learnt a lot and grown… The #FTM thing is very it's very trendy, it's relating to teens as well. So, I really enjoyed it …”* (F4, RC, female). Another added, *“… The programme itself is very well put together … well-researched, and the content is really amazing … the way that the lessons have been planned and the way that the content builds on top of the previous session, I think is great …”* (F2, RC, F).

Most adolescent participants also spoke to their general sense of enjoyment of the programme. They expressed their appreciation for the programme, and their beliefs that the programme was impactful. One noted, *“I was like, why does this thing have to end? It was something I was enjoying… You guys are changing many teenagers' lives … Thank you from the bottom of my heart…”* (P11, 17, F). Many also expressed strong support for expanding the programme to more schools, for all learners, with one principal noting that it would be *“a crucial component for any school”* (CS5, school principal, M). Similarly, CS2 (SW, F) expressed, *"[…] I wish we could go to more schools… it would be great if more schools could have it."* The accessibility for students through schools was seen as a key advantage, with one counsellor stating, *“… in terms of reaching adolescents, the best way to do it is at school”* (CS1, RC, F).

#### Acceptability of #FTM facilitators and facilitator characteristics

All groups of participants stressed the need for facilitators to create a safe, non-judgmental space during sessions, and highlighted the value of building personal relationships over the course of the programme. Some emphasized that safe spaces were especially scarce for some adolescents, with an RC noting, "*They didn't feel judged … lots of them don't have that"* CS2 (SW, F). Many adolescents echoed these sentiments about the creation of safe emotional spaces in the programme. One described how it was *"different"* and how facilitators *"really listen to us without judgmentals”* (P9, female, 18). Others likened facilitators to *“our mothers,”* saying, *"You listened to every problem … made it seem like home …"* (P18, 18, F). Some adolescents also appreciated facilitators' care and concern, with one saying, *"You always ask us questions we want people to ask, like ‘are you good today?’"* (P15, 15, F).

### Perceptions of training and supervision

Facilitators reflected on their experiences with the training and supervision processes, emphasizing the overall comprehensiveness of the training and the crucial role of supervision. F3 (SW, F) noted: *“It was a very nice introduction to the programme… it covered a lot… It covered what to do in certain situations, screenings, which was all very important.”* On the other hand, some facilitators expressed a desire for more preparation in managing challenging situations during the screening process. F2 (RC, F) expressed: *“I do think going through the training, a little bit more focus on what to do in various scenarios during screening. Because, we all had a lot of stuff coming up during that process…”* Generally, facilitators appreciated the supportive, flexible work environment and the opportunity to ask questions, as F4 (RC,F) shared, *"It's easy to…raise whatever concerns…The experience…exceeded my expectations…we were allowed to be ourselves and…[make] mistakes… The supervisors, you are able to…engage with them freely without experiencing problems.”*

Facilitators also appreciated the participant-centred pedagogy during training and supervision, which helped them internalize and apply the content. Facilitators generally found supervision helpful for recapping training, receiving feedback and feeling confident to deliver the programme. F3 (SW, F) noted, *“The supervision definitely helps you prepare for facilitating…familiarizing yourself with the content so that when you get to the sessions you are quite comfortable.”* Though challenging, facilitators also valued receiving peer feedback on the audio-recorded sessions. F1 (RC, F) expressed, *"It was useful listening to the other team…you can hear what they're doing well and not so well and apply that to your own sessions."* On the other hand, some facilitators expressed that while the peer-review process was useful for learning, it was also intimidating: “*… it did add a little bit of pressure in the sessions, knowing someone's listening…I didn't quite like that, so perhaps there were certain things I didn't do as I would have done usually, because I knew I was being listened to”* (F3, SW, F).

### Perceived barriers and facilitators to programme delivery and retention

This theme describes the perceived barriers and facilitators to delivery and retention, including: 1) School engagement, 2) Motivation for programme attendance and MH stigma and 3) Logistical factors for programme delivery and retention. This theme also includes suggested recommendations to enhance delivery and retention, addressing some of the barriers described.

#### School engagement

Community stakeholders and facilitators emphasized the importance of securing school leadership and teaching staff buy-in for the programme's success. CS1 (RC, F) noted the value of direct engagement with principals, stating, *“…the principals…need to give the go-ahead…It’s important to involve them…once that happened, it…made things a lot better.”* Stakeholders perceived that this helped to streamline logistics and foster necessary support for implementing the programme. Where teachers supported the programme and found benefit, there were limited barriers to recruitment. However, this was not always the case. Stakeholders in some schools felt that they hadn’t been adequately informed and stressed the need to clarify the programme's aims in greater detail to gain their support for recruitment. CS3 (teacher, F) explained, *“…They know there are people running this programme, but into details, I don't think they have that information.”*

To improve school buy-in, some stakeholders recommended early engagement and clear communication with staff to outlining the programme's goals and benefits. A school principal suggested, *“…come and [say] you're starting it…[Tell them] ‘this is who we are…what we want to achieve…’ [Create] a connect”* (CS7, M). Some participants suggested that equipping teachers with ER skills would better prepare them to support their students and help foster buy-in for the programme. Here, CS3 (teacher, F) recommended, *“…It would be useful for them…to know how to handle these things in class.”*

#### Motivation for programme attendance and MH stigma

Participants noted several factors that were seen to influence programme enrollment and participation, including self-motivation and stigma. Some adolescents cited recognising their need for support and desire for personal growth as motivation for joining the programme. P3 explained, *“I noticed that I actually need it…that’s why I went.”* P17 (17, M) added, *“In order to become a better person, you have to put in the work. So I decided to come.”* By contrast, some male participants mentioned gender-related considerations regarding poor motivation to join MH programmes, with P12 (15, M) noting, *“…Mostly the guys…believe it’s not useful for them.”*

MH stigma from communities, parents and adolescents themselves was described as a barrier to enrollment and attendance. Some stakeholders noted the negative assumptions associated with attending the programme, such as the belief that community stigma can lead people to dismiss adolescent struggles as *“…looking for attention,”* stating, *“…as long as they can't see you physically hurt, it's not a thing for them.”* Several adolescents echoed these concerns, with some highlighting caregiver hesitance as an attendance barrier. P1 (female, 17) noted, *“…Parents just don’t believe in mental health…letting parents know [more] might help.”* P5 (17, M) also highlighted that seeking help often leads to ridicule, particularly for young men: *“…You would generally be made fun of…because they seem ‘weak.’”* To reduce stigma, some adolescents recommended using alternative language and advertising strategies. They suggested replacing “MH” with “wellbeing” on consent forms to change perceptions and utilizing social media to help destigmatize MH.

#### Logistical factors for programme delivery and retention

Important logistical barriers and facilitators were identified by participants, including programme and session timing, the role of transport reimbursements and sustenance during sessions as well as the use of effective communication strategies.

***Programme and session timing***

##### Programme and session timing

Attendance was perceived as being affected by exam pressure and scheduling conflicts. F2 (RC, F) pointed out that many students faced attendance issues because of, *“…matrics [grade 12 s] wanting to focus on exams… and [some participants] chose sport over the programme."* Many community stakeholders and facilitators recommended scheduling the programme at a more suitable time in the school year. As CS1 (RC, F) suggested, *“We need to be aware of the school's timetable… the time of year matters… during exams, we don't want to distract them."*

Regarding session timing, most adolescents found the two-hour session duration suitable for learning, describing it as *“the sweet spot”* (P5, 17, M). However, facilitators also mentioned that some sessions felt rushed, *"There’s too much content being packed into one session"* (F2, RC, F). To address these concerns, facilitators suggested breaking up content-heavy sessions and adding more sessions. Similarly, many adolescents and stakeholders felt that the programme needed more sessions, including a booster session after programme completion to allow adolescents to fully integrate and apply the skills learned.

##### Transport reimbursements and sustenance during sessions

Participants noted that transport reimbursements and food provision were key to maintaining programme attendance. All participant groups noted that transport reimbursement, in particular, was critical, with many adolescents relying on it to attend after-school sessions. Facilitators confirmed this notion saying that some adolescents, *“…definitely wouldn't have been there without the transport money…”* (F2, RC, F). Participants also reported that food provision enhanced their experience and encouraged attendance. Both stakeholders and adolescents expressed satisfaction with the food. Adolescents appreciated the meals, with P19 (17, M) saying, *“You don't want to be at a place whereby you'll be hungry!”.*

##### Communication

Effective communication was identified as a key facilitator for the programme’s success, particularly when engaging with schools and teachers to support effective collaboration and *“clear as daylight”* (P17, 17, M) communication with adolescents. Methods such as emailing, WhatsApp groups, and in-person meetings were reported to work well. CS1 (RC, F) further emphasized the value of WhatsApp groups for keeping adolescents informed, stating, *“The one thing that is helpful is having that WhatsApp group…to remind students where to go and when.”* This platform was praised for streamlining communication and ensuring that adolescents remained well-informed and engaged.

### Impact of the programme

This theme illustrates the perceived impact of #FTM on adolescent participants’ lives. Three subthemes emerged here including 1) positive shifts in mood and behaviour, 2) salient ER programme skills and perceived benefits and 3) additional benefits.

#### Positive shifts in mood and behaviour

Community stakeholders observed positive shifts in adolescents' emotional well-being after the programme, noting that many continued applying the skills, learned to manage their emotions, avoid harmful behaviours, and become more self-reliant. Improvements in confidence, mood, and social engagement were widely reported. CS6 (SW, F) described a marked change, saying most adolescents *“…commented on it helped them to cope with their emotions… I can see how vibey they are… they shine more now… they're happier, more confident… like their whole personalities have changed!”* Adolescents echoed these sentiments, sharing how the programme helped them improve emotional well-being. P11 (17, F) reflected, *“…before I didn't know how to handle stress. But now I can… You guys don't know what this thing really mean to me… The person that I am today, it's also because of this programme."*

Some adolescents reported specific improvements in mood and behaviour. P18 (17, F) expressed, “…*it gave me more positivity for dealing with life…I just keep moving forward…I’m constantly happy… Everyone’s noticing it. I don’t have time to be overthinking…after doing the sessions, it gave me hope.”* Many participants also noted behavioural improvements, particularly in managing anger. P2 (15, F) shared, *“…I have a short temper…before, I used to get angry all the time. But now, I just breathe in and out. I haven’t been angry in a long time.”* Similarly, P17 (17, M) reflected: *“Before, I would just jump in and start fighting… but now, I calm things down… I’ve learned to let the anger flow out without hurting anyone… Even my behaviour has changed.”*

#### Salient ER skills and perceived benefits

This sub-theme presents participants’ perceptions of salient ER skills and strategies learned through the programme. Salient sub-skills included *Relaxation, Acceptance, Self-support, and Investigation skills,* while salient strategies included all learned ER strategies as well as physical activity.

##### Salient ER subskills: relaxation, acceptance, self-support and investigation

Adolescents acknowledged the value particular sub-skills learned through the programme. Many adolescents emphasized the importance of the *Relaxation* techniques, particularly breathing techniques, in managing stress and improving focus, while progressive muscle relaxation was less popular. These skills also enhanced other coping strategies learned in the programme. For example, P17 (17, M) noted, *"Relaxation…gives you time to rest and figure things out…The five-star method…calmed me down." Acceptance* was seen as an essential but challenging skill for adolescents, as it required learning to tolerate emotions rather than reacting impulsively. Others stressed the importance of recognizing feelings as normal and functional, as P13 (15, F) explained, *“[When I feel angry] I tell myself…I’m allowed to feel this way…I realized you should tolerate the feeling…It has a message*.” Some participants also reflected on learning to accept life’s challenges. Here P19 (17, M) expressed: *“I learned that there are certain things in life that won’t work out…And I have to accept…it’s like that.”* The *Self-support* module, which focused on enhancing self-esteem, was also perceived as beneficial. P13 (15, F) said, *“…I sometimes look in the mirror and I'm like, ‘girl, you have 11 certificates…You are very smart. You are your mom's daughter!’”* Similarly, P14 (15, F) noted, *“We saw ourselves in a more positive way…Self-support really helped us… [with] more confidence…We thought positive about ourselves more than before the programme.”* Finally, many participants also highlighted the value of the *Investigation* skills, which helped them better understand links between feelings, thoughts, and actions. For example, P14 (15, F) elaborated on how she now reflects on her feelings and the consequences of her actions:

*I didn’t know you can investigate your feelings. Now I do that a lot... I ask myself, ‘how am I gonna see this situation?’ It’s really helpful because a lot of teenagers just don’t think about the consequences, they just do things... Especially with feelings, like with boys and stuff.*

##### ER strategies: distraction, problem solving, thinking differently

Adolescents found all three ER strategies helpful, though some were more challenging than others. *Distraction* was the most popular and easiest to apply. Many adolescents used activities like dancing or listening to music to manage stress. Participants found *Problem-Solving* valuable for emotional management and conflict resolution. P18 (17, F) reflected, *“…some information you guys told me, I never actually knew…problem solving…Especially the one where you know how to control your feelings, come to solutions…”* P15 (15, F) also described how she had applied this strategy to resolve conflicts: *“…if one of my friends…argues…I just say, ‘wait…Let’s sit and talk…see where we went wrong…then…forgive each other.’”* The *Thinking Differently* strategy was perceived as most challenging. Facilitators acknowledged this difficulty, as F1 (RC, F) noted, *“Look, it is a tricky one, so it will just take practice.”* Despite the challenge, P16 (17, F) appreciated its impact: *“…if I didn't come to this programme, I wouldn't have learned that I must change the way I think.”* Some adolescents also shared how *Thinking Differently* helped them embrace positive thoughts and gain perspective on stressful situations. For example, P1 (17, F) expressed, *“Thinking differently…makes you see things from a different point of view…It helps you relax a lot and feel better about the situation.”*

##### Physical activity

Many adolescents emphasized the importance of the physical activity elements in the programme, particularly as stakeholders highlighted the increasing sedentary lifestyle among teenagers. CS2 (SW, F) remarked that *“…today’s generation…exercise is now consumed with a screen.”* Some facilitators noted initial resistance to physical activity among certain adolescents, which later transformed into appreciation as the programme progressed. This shift in perspective was echoed by several adolescents, including P11 (17, F): *“The gymming club…at the beginning, it was really hard…then I started to enjoy it…listening to music…was relieving.”* Several adolescents reported integrating physical activity into their daily lives post-programme. P17 (17, M) related that he now sets physical activity goals for himself, *“Like you said in the programme, set yourself a target every day…yesterday, my target was 30,000 steps… When I got on the bus, I reached my goal…I'm basically using the skills every day.”* Establishing consistent exercise routines was seen as beneficial for a variety of reasons, including reflection and processing, and stress management. P19 (17, M) noted, *“I take walks…every day…whilst being on that walk I reflect on my day…ways that I could be still with things that I don't like.”* P16 (17, F) explained how PA affected her wellbeing:

*...before I wasn't a person that used to move my body a lot...it pushed me to move my body more. I could see the difference…when I exercise, I feel like there's this load that's taken off me…if I'm stressed...I exercise, and then...I'll feel relaxed a bit.*

Adolescents and facilitators alike highlighted physical activity’s role in enhancing engagement and content understanding. Participants described how physical activity functioned as an energizer, sustaining attention and motivation, after long school days. F1 (RC, F) observed, *"I see their energy drop…when they did the PA, it’s amazing! Then they're back on track.”* It helped some learners grasp ER content through simple, practical methods, making it easier to apply skills to their own emotional processes. For example, P12 (15, M) expressed that he appreciated learning through physical activity because, *"…the person who is learning these skills is actually feeling something in the moment to be able to put the skills to use or to know how to use it. So…it was a good thing that it was linked."* Facilitators echoed sentiments about using physical activity as a learning vehicle. F2 (RC, F) remarked, *“…I think the way it was linked to the modules was fantastic […] physical activity helps you to really learn something. It makes it easier and more fun to learn.”*

#### Additional benefits

Additional benefits described by participants included the indirect positive impact of the programme on adolescents’ approach to academics; and fostering new friendships and sense of belonging.

##### Positive impact on approach to academics

Some adolescents shared how the programme positively impacted their ability to manage academic stress, boost academic confidence, and improve academic performance. Here, P18 (17, F) described her exam experience after completing the programme, saying, *“…everything was fine. I was calm. I wasn't like, bottling up like, [thinking] ‘this paper is hard…’.”* P20 (16, F) shared a similar experience, stating, *“…I was stressed, but I said to myself, I can do it to go through the grade.”* As her progress improved, she noted, *“And now, I'm getting better and better,”* attributing her success to using the programme’s steps: *“‘Cos why? I'm using the FTM programme steps in my work.”* Others spoke about overcoming negative thoughts regarding their academic abilities. For example, P11 (17, F) mentioned, *“I was thinking of leaving [school], but I didn’t allow those negative words to work on me.”* Instead, she used confidence boosters from the programme, telling herself, *“You can do it. You can make it…after the programme, I managed to handle it.”* She felt proud when her hard work paid off: *“…the moment I got my result, everything went good. I have improved.”*

##### Fostering new friendships and sense of belonging

Many participants noted that the programme helped reduce feelings of social isolation and that they valued the sense of belonging, friendship, and emotional support it fostered among peers. Here, P1 (17, F) expressed, “*We were all very sad when it ended. We actually made new friends.”* Similarly, P19 (17, M) stated, *“…After the programme, I missed…the people…I used to hang out with.”* Some also noted how the programme helped them feel socially connected, allowing them to bond over shared experiences as described by CS2 (SW, F): *“It felt like a little family….”* P2 (16, F) echoed this, sharing her growth in building friendships: *“I learned how to start relationships…We’re no longer strangers.”*

### Perceived facilitators of learning

This theme presents two key factors that supported learning: 1) experiential learning and the importance of having fun, and 2) #FTM Materials, including digital and hardcopy resources.

#### Experiential learning and the importance of having fun

Participants appreciated the practical, creative, and engaging ways knowledge was taught in the programme, with both facilitators and adolescents valuing experiential learning over passive methods. As F1 (RC, F) noted, *“…Hands-on learning. I think they enjoy it most.”* Adolescents shared this view, with P2 (15, F) saying she preferred practical learning to *“just sitting and writing stuff.”* P1 (16, F) also described how integrating multiple forms of experiential learning—through physical activity, writing, and watching videos—kept adolescents stimulated and contributed to their overall learning.

Having fun was also crucial in enhancing adolescents’ learning experiences. Engaging activities, games, and listening to music were key to creating a pleasant and refreshing environment. P5 (17, M) expressed his surprise at how much fun he had, saying, *“I didn’t expect the programme to be so fun… I thought it was going to be serious.”* Facilitators highlighted how fun activities sustained motivation, with F2 (RC, F) noting, *“…when there [are] these refreshing new ways, they want to come and the time flies by!”.*

#### #FTM digital and hardcopy materials

Participants valued digital and hardcopy materials as tools for learning and engagement. Digital resources like audio recordings and psychoeducation videos enhanced skills and provided visual demonstrations for complex concepts. Facilitators noted that these resources allowed adolescents to practice outside sessions: *“…you could hear through feedback that…they’re enjoying this. It’s actually helping them”* (F4, RC, F). P14 (15, F) highlighted how videos made learning easier: *“…with the booklet, you’ll have to read, but…if you listen to it…see movements, it’s very helpful…because…a lot of kids don’t really understand…[So] the videos and the actions…those demonstrations were very helpful.”* Similarly, P11 (17, F) explained how the videos improved participation in sessions: *“…after watching the video, then it would be easier for us to give an answer…it makes it easier for us to understand.”*

Hardcopy materials, like activity books and puzzles, also supported learning retention and emotional connection to the content. P11 (17, F) shared, *“…I still have the book…sometimes I go through the book, check the exercise and do it myself…I still have that [puzzle pieces], I’ve pasted it on my fridge…It brings nice memories back.”* Visual reminders reinforced stress management techniques, such as P14 (15, F) practicing when stressed: *“…when I’m stressed out…I go to the fridge and practise some, depending on the situation.”* The materials’ presence strengthened adolescents’ connection to the content, as P16 (17, F) explained *“I like the star breathing…because I still have the picture…it has indications for inhaling and exhaling.”* Some adolescents also found the recordings calming and reflective, as P11 (17, F) shared, *“…you get…to just relax, thinking about things…letting that thing come into your mind…It’s only yourself there. There’s peace. All of us enjoyed that.”*

## Discussion

Despite the wealth of literature on school-based interventions, the evidence for task-shared programmes in low-resourced settings is relatively scarce, with even less data available on participants’ perceptions of such interventions. This study explored perceptions of a school-based ER intervention with physical activity elements for older adolescents in low-income settings, specifically the acceptability, feasibility, and perceived impact of the task-shared programme. Key findings discussed below include perceptions of the programme, and training and supervision; barriers and facilitators to programme success; positive programme impact; salient skills learned and important facilitators of learning.

Participants found the programme acceptable, enjoyable, and necessary for addressing adolescents' MH needs, aligning with findings from similar interventions in South Africa and comparable contexts. The interventions below also include teaching ER, problem-solving and stress management skills, which seem to have been well received by participants. For instance, the "Four Steps To My Future" programme was considered relevant and enjoyable [74], while the "ASPIRE" intervention was valued for its cultural appropriateness and supportive environment [75]. The "HASHTAG" programme in South Africa and Nepal was also seen as culturally relevant and feasible, engaging stakeholders, addressing stigma, and supporting ER [76]. Given that programme acceptability and enjoyment are key predictors of success and positive outcomes in adolescent psycho-social interventions, it is vital that programmes are contextually appropriate and engaging [77–81].

Participants valued facilitators for creating a safe, compassionate environment. Selecting and training facilitators who can effectively build therapeutic relationships is essential for the success of psycho-social interventions [67, 82]. Incorporating key competency items in the TACT-Groups-SA on facilitator warmth, non-judgement, playfulness, and participant affirmation likely aided in fostering this perceived safe space in the #FTM intervention [83]. Qualitative evidence on task-shared adolescent MH interventions further highlights the critical roles of trust, reliability, and non-judgmental approaches in building strong therapeutic alliances, which are key to successful interventions and improved MH outcomes [84, 85]. As such, high-quality facilitator training should be an implementation focus to improve programme acceptability and possible impact.

Facilitators found the training and supervision processes acceptable, with the competency-based approach being particularly useful. A task-sharing model, adapted from previous South African research, combined comprehensive training with ongoing and ad hoc supervision [68, 69]. Facilitator competency was assessed and monitored using the TACT-Groups-SA, adapted from the ENACT, WE-ACT and Group-ACT tools (see footnote^4^) [67, 86]. Competency-based approaches have been shown to be more effective than traditional methods in enhancing facilitator skills in such settings [83, 87]. Further, utilising competency-based approaches in training and supervision is well received by facilitators and crucial for task-shared models [88–90], highlighting the need for competency-driven training to support feasibility and sustainability in low-resource settings [91–94].

Participants identified key barriers and facilitators to programme delivery and retention, including school engagement, motivation, MH stigma, and logistical issues. Systematic reviews of school-based MH interventions highlight the importance of school-stakeholder engagement through collaborative planning, alongside participant motivation and consistent communication, for success and sustainability [95–97]. Research on adolescent MH programme access highlights several barriers, including stigma, limited awareness of MH issues and available services, logistical constraints (transportation and scheduling difficulties), and competing academic or social commitments [91, 98, 99]. While local studies on task-shared interventions remain limited, research from sub-Saharan Africa identifies similar challenges, including stigma, the need for relevant training, and the importance of community engagement to improve programme uptake [98, 100, 101]. These findings emphasize the need to address these factors to improve programme delivery and retention in school settings.

The #FTM programme was perceived as having positively impacted participants through the application of ER skills and strategies and the use of physical activity. The positive impacts described included improvements in mood, behaviour, anger control, and reductions in stress, anxiety, and depression symptoms. These findings align with quantitative research linking improved ER to better MH among adolescents [19, 33, 102–105]. Our findings regarding improved anger management align with findings from a similar task-shared ER intervention for adolescents in Nepal [106]. The positive shift in anger control is particularly relevant in South Africa, where high rates of interpersonal violence among young people present significant public and MH concerns [107]. Anger management difficulties are closely linked to poor ER in adolescents [108], and given that intentional injuries contribute significantly to local mortality rates [109], equipping adolescents with effective anger management skills is crucial for improving MH outcomes.

Key subskills, such as Relaxation, Awareness, and Acceptance, were seen as particularly beneficial. Relaxation techniques like deep breathing reduce stress by calming physiological responses [110]. Awareness aids adolescents in reflecting before reacting, helping them choose more adaptive responses in stressful situations [111, 112]. Acceptance supports tolerance of uncomfortable emotions, reducing avoidance and internalising symptoms, and promoting emotional adaptability during emotional fluctuation [113]. Although qualitative studies on perceptions of these subskills are limited, similar adaptations of ART [48, 55, 114, 115] and other psychosocial interventions for adolescents [116–118] have found these subskills effective in managing emotions and improving behavioural choices.

Participants found the Self-esteem and Investigation modules highly relevant. Participants highlighted the role of self-esteem in building confidence and helping them feel better, aligning with research indicating that higher self-esteem can reduce the risk of depression and anxiety [119–123]. Although qualitative research is limited, studies suggest that adolescents report gaining self-confidence through similar psycho-social programmes that incorporate self-esteem components [74, 124]. Participants also found the Investigation module valuable in understanding the links between feelings, thoughts, actions, and consequences. Grounded in Cognitive Behavioural Therapy, such Investigation skills are known to promote adaptive coping strategies and improve MH outcomes among adolescents [102, 125]. By helping adolescents recognize that these elements of experience are interlinked and modifiable, these skills can help adolescents take a more active role in shaping their emotional experiences. These findings align with similar studies showing positive impacts of these subskills on MH, though qualitative data is limited [48, 53, 55, 114, 115].

Adolescents found ER strategies like Distraction, Problem Solving, and Cognitive Reframing useful, though some were more challenging than others. While quantitative research shows these evidence-based strategies improve ER and mood [126–129], qualitative data on participant perceptions of these strategies are limited. Existing research suggests adolescents view strategies like cognitive reappraisal, problem solving, and distraction as helpful for coping and making rational, adaptive decisions [118, 124, 130]. Emerging concepts like polyregulation (the use of multiple ER strategies, simultaneously or sequentially, to alter emotional outcomes) [131] and ER flexibility (adapting strategies based on context and goals) [132, 133] highlight the importance of adaptable ER skills, crucial during adolescence to foster emotional resilience, improve MH, and support long-term well-being through challenges and into adulthood.

Participants emphasised the value of physical activity for emotional coping despite initial resistance from some adolescents. Though limited, local qualitative research shows similar findings, with adolescents in a skateboarding intervention reporting that physical activity provided opportunities to relieve stress and cope with strong emotions [57]. International qualitative research on adolescents' perspectives also reflects a preference for locally accessible, low-cost, and enjoyable physical activity opportunities that prioritize social interaction, engagement and flexibility in physical activity options [134]. Quantitative research demonstrates that physical activity counteracts sedentary lifestyles linked to poor MH outcomes like anxiety and depression in adolescence [135–139] reducing stress and strengthening neural circuits involved in emotional awareness and control [30, 46, 138, 140]. Establishing adaptive physical activity habits during adolescence can offer long-term health benefits and boosts emotional resilience [139, 141].

Participants also highlighted additional programme benefits, positive impact on approach to academics and new friendships, and a sense of belonging. Participants also expressed perceptions of the positive impact of the programme on their approach towards academics. While qualitative research in this area is limited, available research shows strong associations between adaptive ER skills and improved academic stress management and academic performance [142, 143]. The findings are noteworthy given that academic achievement significantly impacts career and MH trajectories, especially for adolescents from low-income settings facing multiple barriers to academic achievement [144, 145]. Additionally, participants valued the sense of belonging and peer connections fostered through the programme, consistent with quantitative and qualitative evidence that structured group activities enhance social skills, reduce perceptions of isolation, and promote perceptions of social connectedness [84, 124, 146–149]. These findings are significant, as loneliness is a key risk factor for depression and anxiety, and group-based interventions may help mitigate this risk [150–152].

Finally, participants viewed experiential learning, enjoyable activities, and access to both digital and hardcopy materials as key facilitators of learning that enhanced their perceptions of the programme. For instance, #FTM adolescent participants reported that physical activity helped them better understand the ER concepts, aligning with research showing its usefulness as a pedagogical tool for grasping abstract ideas and supporting skill retention in real-life contexts [153]. While qualitative research on adolescents' perceptions of experiential learning in such programmes is limited, meta-analytic studies highlight its role in fostering participation, motivation, and understanding, while fun activities promote engagement and feelings of comfort and ease [154, 155]. A recent scoping review similarly advocates for incorporating social and leisure activities in adolescent programmes [156]. Participants also found the digital and hardcopy materials to be important for their learning journeys. Digital resources, such as audio and video materials, support diverse learning styles and reinforce skills, while hardcopy materials serve as tangible reminders to integrate skills into daily life [157, 158]. Qualitative data on these aspects are limited, underscoring the need to explore how these learning facilitators contribute to programme success and MH outcomes for this unique population.

This study has several limitations. The findings may not be fully generalizable, though aspects of #FTM and the research process may inform future studies in similar settings. Researcher subjectivity could impact objectivity, though this was mitigated by co-developing the coding framework and using multiple coders. Member-checking of transcripts was not conducted, which may have resulted in the researchers’ interpretation not fully aligning with the meaning assigned by the participants. The sample had fewer males than females, reflecting similar lower male participation in MH studies [159], often due to stigma and masculinity-related barriers [99, 160]. Participants who exited early were not interviewed, potentially excluding valuable perspectives. Despite these limitations, the study offers key insights into the programme’s feasibility, acceptability, and perceived impact, laying the groundwork for future research and improvements in similar contexts.

## Conclusion

This study is one of the first to explore participants’ perceptions of an innovative school-based, indicated ER intervention with engaging physical-activity elements for older adolescents from low-income backgrounds. The findings support the feasibility and acceptability of the task-shared intervention while also demonstrating participants’ perceptions of the positive impact and benefits of the programme. This research highlights the value of gaining in-depth insights from multiple stakeholders about such interventions and their potential to improve adolescent MH outcomes. It underscores the potential of creative, experiential, evidence-based ER and physical activity approaches in addressing adolescent MH needs in low-resource settings.

## Supplementary Information

Supplementary Material 1.

Supplementary Material 2.

## Data Availability

Data is managed in accordance with the University of Cape Town’s Intellectual Property and Research Data Management Policies. Databases are stored on the University of Cape Town’s firewall-protected server. Hard-copy data is securely kept in locked CPMH cabinets for five years before destruction, accessible only to the lead researcher and team. De-identified qualitative transcripts are stored on password-protected laptops. With permission, quantitative data will be stored indefinitely in the CPMH registry on ZivaHub and shared upon request via CPMH processes, following the Human Research Ethics Committee guidelines.

## Abbreviations

ART: Affect Regulation Training
CS: Community Stakeholder
ENACT: ENhancing Assessment of Common Therapeutic factors for group facilitation
ER: Emotion Regulation
EQUIP: Ensuring Quality in Psychological Support guidelines
F: Female
#FTM: #FeelThinkMove
Group ACT: Adolescent Group Competencies Training
M: Male
MHC(s): Mental Health Condition(s)
MH: Mental Health
P: Participant
PTSD: Post-Traumatic Stress Disorder
RC: Registered Counsellor
SW: Social Worker
YLD(s): Years Lived with Disability
WE ACT: Wellness and Empowerment through Active Competencies Training for Adolescents

## Footnotes

1 Note that these estimates are based largely on symptom prevalence screening results rather than diagnostic interview results.

2 https://communitykeepers.org/.

3 Adolescent participants were excluded if they were currently receiving psychological treatment elsewhere or if they were actively suicidal. In the latter case, participants were referred immediately for assessment and treatment by a CK counsellor.

4 The TACT-Group-SA was adapted using the following WHO- and UNICEF-supported‘Ensuring Quality in Psychological Support’ (EQUIP) guidelines: the ENhancing Assessment of Common Therapeutic (ENACT) factors for group facilitation, the Wellness and Empowerment through Active Competencies Training for Adolescents (WE ACT), and the Adolescent Group Competencies Training (Group ACT) (Kohrt et al., 2024; Kohrt et al., 2015).
