# Supplementary material for: Perceived feasibility and acceptability of an innovative emotion regulation programme with physical activity elements for older South African adolescents from low-income settings: a qualitative study
Source: BMC Pediatr. 2025 Nov 11;25:921. doi: 10.1186/s12887-025-06280-6 (PMC12606897; doi:10.1186/s12887-025-06280-6)
Supplement: Supplementary file 1 — Supplementary Material 1. [file 12887_2025_6280_MOESM1_ESM.docx]

| **Topic** | **Item No.** | **Guide Questions/Description** | **Reported on Page No.** |
| --- | --- | --- | --- |
| **Domain 1: Research team and reflexivity** | | |  |
| ***Personal characteristics*** | | | |
| Interviewer/facilitator | 1 | Who conducted the interviews? | 6-7 |
| Credentials | 2 | What were the researcher’s credentials? E.g., PhD, MD | 6-7 |
| Occupation | 3 | What was their occupation at the time of the study? | 6-7 |
| Gender | 4 | Was the researcher male or female? | 6-7 |
| Experience and training | 5 | What experience or training did the researcher have? | 6-7 |
| ***Relationship with participants*** | | | |
| Relationship established | 6 | Was a relationship established prior to study commencement? | 6 |
| Participant knowledge of the interviewer | 7 | What did the participants know about the researcher? e.g., personal goals, reasons for doing the research | 6 |
| Interviewer characteristics | 8 | What characteristics were reported about the inter viewer/facilitator? e.g., Bias, assumptions, reasons, and interests in the research topic | 6 |
| **Domain 2: Study design** | | | |
| ***Theoretical framework*** | | | |
| Methodological orientation and Theory | 9 | What methodological orientation was stated to underpin the study? e.g. grounded theory, discourse analysis, ethnography, phenomenology, content analysis | 10 |
| ***Participant selection*** |  |  |  |
| Sampling | 10 | How were participants selected? e.g., purposive, convenience, consecutive, snowball | 5-6 |
| Method of approach | 11 | How were participants approached? e.g., face-to-face, telephone, mail, email | 6 |
| Sample size | 12 | How many participants were in the study? | 6-7 |
| Non-participation | 13 | How many people refused to participate or dropped out? Reasons? | 6 |
| Setting |  |  |  |
| Setting of data collection | 14 | Where was the data collected? e.g., home, clinic, workplace | 6-7 |
| Presence of nonparticipants | 15 | Was anyone else present besides the participants and researchers? | 6-7 |
| Description of sample | 16 | What are the important characteristics of the sample? e.g., demographic data. | 11 |
| Data collection |  |  |  |
| Interview guide | 17 | Were questions, prompts, guides provided by the authors? | 6-7, 46-49 |
| Repeat interviews | 18 | Were repeat interviews carried out? If yes, how many? | N/A: No repeat interviews were carried out |
| Audio/visual recording | 19 | Did the research use audio or visual recording to collect the data? | 6 |
| Field notes | 20 | Were field notes made during and/or after the interview or focus group? | N/A: No field notes were captured due to the audio recordings capturing the necessary data |
| Duration | 21 | What was the duration of the interviews or focus group? | 6 |
| Data saturation | 22 | Was data saturation discussed? | 6 |
| Transcripts returned | 23 | Were transcripts returned to participants for comment? | 28 |
| Topic | Item No. | Guide Questions/Description | Reported on Page No. |
|  |  |  |  |
| **Domain 3: analysis and findings** | | | |
| ***Data analysis*** |  |  |  |
| Number of data coders | 24 | How many data coders coded the data? | 10 |
| Description of the coding tree | 25 | Did authors provide a description of the coding tree? | 11-12 |
| Derivation of themes | 26 | Were themes identified in advance or derived from the data? | 10 |
| Software | 27 | What software, if applicable, was used to manage the data? | 10 |
| Participant checking | 28 | Did participants provide feedback on the findings? | 28 |
| Reporting |  |  |  |
| Quotations presented | 29 | Were participant quotations presented to illustrate the themes/findings?  Was each quotation identified? e.g., participant number | 12-23 |
| Data and findings consistent | 30 | Was there consistency between the data presented and the findings? | 12-23 |
| Clarity of major themes | 31 | Were major themes clearly presented in the findings? | 24-28 |
| Clarity of minor themes | 32 | Is there a description of diverse cases or discussion of minor themes? | 24-28 |
