# Supplementary material for: Perceived feasibility and acceptability of an innovative emotion regulation programme with physical activity elements for older South African adolescents from low-income settings: a qualitative study
Source: BMC Pediatr. 2025 Nov 11;25:921. doi: 10.1186/s12887-025-06280-6 (PMC12606897; doi:10.1186/s12887-025-06280-6)
Supplement: Supplementary file 2 — Supplementary Material 2. [file 12887_2025_6280_MOESM2_ESM.docx]

# Adolescent interview guide

**Hello and thank you for taking the time to answer some feedback questions about your experience with the #FeelThinkMove (#FTM) programme. Please be as open as possible as your feedback will help us to improve the programme for future learners.**

**The #FTM Programme**

**First, I want to ask you some questions about the #FTM Programme itself….**

1. What did you think about the #FTM Programme in general? **Probes**: Did you enjoy it? Why or why not? Please elaborate here. Was it what you expected?
2. What did you think about the #FTM content, i.e., what you learned in the programme? **Probes**: emotion regulation skills: *relaxation* (belly and star breathing, muscle relaxation), *awareness* (4 steps to awareness), *acceptance* (allowing and tolerating), *self-support* (self-esteem, self-care, positive self-talk), investigation (the 5 components of investigation) emotion regulation strategies (distraction, problem solving, thinking differently); and ***physical activity*** (gym club, other physical activities done in session).
3. Are there any #FTM modules/skills/activities that worked really well/that you liked **or** that need to be changed/modified/that you didn’t like? (See probes as per question 2).
4. If you had to choose, what do you think the most important skill/part from the programme was for you/adolescents? Why?
5. If you had to choose, what do you think the least important skills/parts of the programme were for you/adolescents? Why?
6. What did you think about the physical activity parts of the sessions? **Probes**: please explain what worked well and what did not work well, and how could we improve this element of the programme?
7. When you participated in the programme, what, if any, positive changes did you notice in yourself or others from the group? **Probes**: did you notice any negative changes in yourself or others in the group because of the programme as well? Please elaborate.

**Logistics questions**

**I’d like to ask you some questions about the logistics of the programme now….**

1. Did the programme take place at a good time during the year, why/why not?
2. Did the programme take place at a good time during the day, why/why not?
3. Did the programme last a good amount of time each session (i.e., 2 hours per session)?
4. What did you think about the length of the programme as a whole (i.e., 7 weeks long)? Probes: do you think the programme should be longer, shorter or the same number of sessions, and why?
5. What was attendance like in your sessions? **Probes**: did the majority of participants show up for sessions or not? Were there any barriers (strike action for example) or motivational factors that made attendance better (transport cash)?
6. What are your thoughts about having an additional ‘booster’ session/s a few months after the programme has ended? Why/why not?
7. Did anyone from your group drop out of the programme? If yes, why do you think participants dropped out? Did you ever want to drop out of the programme, if yes, how come?
8. Do you have any recommendations on how we could improve attendance if we were going to roll out this programme in future? Please elaborate.
9. Are there any other logistical issues that need improvement? For example, issues with transport money, session timing, session duration, programme duration or food or anything else not mentioned etc? please elaborate.

**Final thoughts**

1. Do you have any other comments for us about any aspect of #FTM, or any other ways you think we can make it better for future learners?

**THANK YOU (give voucher and sign voucher receipt form)**

# Facilitator interview guide

**Hello and thank you for taking the time to answer some feedback questions about your experience with the #FeelThinkMove (#FTM) programme and the training and supervision processes. Please be as open as possible as your feedback will help us to improve the programme, training, and supervision processes.**

1. **Why did you first apply to become an #FTM facilitator?**

**The #FTM Programme**

**First, I want to ask you some questions about the #FTM Programme itself….**

1. What did you think about the #FTM Programme in general? **Probes**: Did you find it to be an acceptable programme for our context…what did you think of the length of the programme? Please elaborate here.
2. What did you think about the #FTM content? **Probes**: emotion regulation skills: *relaxation* (belly and star breathing, muscle relaxation), *awareness* (4 steps to awareness), *acceptance* (allowing and tolerating), *self-support* (self-esteem, self-care, positive self-talk), investigation (the 5 components of investigation) emotion regulation strategies (distraction, problem solving, thinking differently); and ***physical activity*** (gym club, other physical activities done in session).
3. Are there any #FTM modules/skills/activities that worked well **or** that need to be changed/modified? (See probes as per question 2).
4. If you had to choose, what do you think the most important aspect of the programme was for participants? Why?
5. If you had to choose, what do you think the least important aspect of the programme was for participants? Why?
6. What did you think about the physical activity parts of the sessions? **Probes**: please explain what worked well and what did not work well, and how could we improve this element of the programme?
7. When adolescents participate in the programme what positive changes can you did you notice in them? **Probes**: did you notice any negative changes in participants as well, please elaborate?

**Logistics**

**I’d like to ask you some questions about the logistics of the programme now….**

1. Can you tell me a little bit about the role of the schools and/or CK in the FTM programme? **Probes**: did you experience any barriers or factors that helped the process along from the schools or CK?
2. What was attendance like in your sessions? (Just in terms of participants showing up for sessions). **Probes**: were there any barriers (strike action for example) or motivational factors that made attendance better (transport cash)?
3. Did you have any dropouts from your groups? If yes, why do you think participants dropped out?
4. Do you have any recommendations on how we could improve attendance if we were going to roll out this programme?
5. Are there any logistical issues that need improvement? For example, issues with transport money, session timing, session duration, programme duration or food or anything else not mentioned?

**Training and supervision**

**Next, I’d like to ask you about your experience of the training and supervision processes:**

1. What did you think of the #FTM training process during that first week you were trained? **Probes**: what worked well for you…. what didn’t work for you about the process? What aspects need some improvement?
2. Was the 40-hour training sufficient? **Probes**: were there any areas of the training that you needed more training in? Did you feel prepared enough for your role as a facilitator/co-facilitator once you were running the groups?
3. What did you think of the facilitator manual? **Probes**: thoughts on the manual structure and layout? How could we improve the manual?
4. What did you think about group supervision and roleplay sessions? **Probes**: what did you like about the process? What aspects of the process didn’t work well for you and how can we improve on these?
5. What did you think of the peer-review process of supervision (rating each other using the TACT and checklists and receiving feedback about this)? **Probes**: what worked well and what didn’t?

**Final thoughts**

1. Do you have any other comments for us about any aspect of #FTM, the support you received, or your experiences of being involved in the programme?

**THANK YOU (give voucher and sign voucher receipt form)**

# Community stakeholder interview guide

**Hello, and thank you for taking the time to answer some feedback questions about your experience with the #FeelThinkMove (#FTM) programme at your school. Please be as open as possible as your feedback will help us to improve the programme and how it is run in the future.**

**General questions**

1. What do you think some of the challenges are that adolescents are facing in terms of their mental health? What are they struggling with mentally? **Probes**: depression, anxiety, substance use, peer pressure, bullying, trauma and violence, teen pregnancy.
2. I’d like to talk a little bit more about an important concept called ‘emotion regulation,’ which is our ability to manage difficult feelings using healthy coping strategies. For example,
3. Learning how to breathe calmly in order to **relax** ourselves.
4. Learning how to **accept how we feel** without judgement in order to move through it.
5. Learning how to **talk to ourselves positively** in order to feel better.
6. Learning how to practice some **self-care** when we are feeling burned out.
7. Being able to **exercise** (like going for a run for example), when we feel stressed in order to feel better.

Do you think these skills are being addressed/taught in school to adolescents? Please explain further.

1. How important do you think these skills are for older adolescents? Why do you think they are important or not?
2. Tell me about how your collaboration with #FTM started? Why did you first decide to collaborate with UCT for the #FTM programme?

**#FTM Programme questions**

**Next, I want to ask you some questions about the #FTM Programme itself….**

1. What do you know/remember about the #FTM programme and its content? **Probes**: To remind you, the #FTM programme is for older adolescents who are experiencing some symptoms of depression and anxiety. The programme is all about helping adolescents to learn specific skills to manage difficult feelings and situations. The programme also included physical activity as we know it is vital for improving mental health.
2. What did you think about the #FTM Programme in general? **Probes**: Did you find it to be an acceptable programme for your context…what did you think of the length of the programme? How appropriate was the content for what the adolescents need at this time in their lives? Please elaborate here.
3. What did you think about the #FTM content itself? **Probes**: for example, teaching adolescents to relax their breathing and their bodies, teaching adolescents how to become aware of, and to accept what they are feeling, teaching them how to take care of and support themselves properly, teaching them specific emotion coping strategies and the importance of physical activity?
4. Were there any particular #FTM skills or activities (from above) that you think worked well **OR** that need to be changed/modified?
5. What did you think about the physical activity aspect of the sessions? **Probes**: do you think physical activity is important for learners for their mental health. Please elaborate.
6. What did you think about the duration of the programme (i.e., 7 sessions)?
7. And what did you think about the length of the sessions (2 hours each once per week)?
8. When adolescents participated in the programme what, if any, positive changes can you did you notice in them? **Probes**: did you notice any positive or negative changes in participants, please elaborate on these?
9. During the programme or after the programme was complete, was there any feedback from the learners about how they experienced it? If yes, please elaborate.
10. Would you recommend that all learners (not just those with depressive/anxiety symptoms) should participate in the programme if it were available? Please elaborate on your answer.

**Logistics questions**

**I’d like to ask you some questions about the logistics of the programme now….**

1. Can you tell me a little bit about the engagement and communication you had with UCT/Chesney/the facilitators? **Probes**: was there anything that made the process of communication/working together easier or harder for you? For example, was there sufficient communication about the programme plan between you and the UCT contacts? Can you think of any tips to improve engagement and communication between you and UCT contacts?
2. What was the principal’s/staff members’ response to the programme? Do you think they bought into the idea of the programme/thought the programme was a good idea? How could we improve their buy-in for the programme in future?
3. Do you have any tips on how to improve learner attendance for the programme in future? **Probes**: for example, would it be better to have the programme during school, could we improve the food or incentives or could we improve the advertising of the programme itself before it starts etc?
4. Are there any logistical issues that need improvement? For example, issues with transport money, session timing, session duration, programme duration or food or anything else not mentioned?

**Final thoughts**

1. Do you have any other comments for us about any aspect of the #FTM programme or anything else logistical or otherwise?

**THANK YOU (give voucher and sign voucher receipt form)**
